# Two Regulators of *Vibrio parahaemolyticus* Play Important Roles in Enterotoxicity by Controlling the Expression of Genes in the Vp-PAI Region

**DOI:** 10.1371/journal.pone.0008678

**Published:** 2010-01-13

**Authors:** Toshio Kodama, Kazuyoshi Gotoh, Hirotaka Hiyoshi, Mikiharu Morita, Kaori Izutsu, Yukihiro Akeda, Kwon-Sam Park, Vlademir V. Cantarelli, Rikard Dryselius, Tetsuya Iida, Takeshi Honda

**Affiliations:** 1 Department of Bacterial Infections, Research Institute for Microbial Diseases, Osaka University, Suita, Osaka, Japan; 2 International Research Center for Infectious Diseases, Research Institute for Microbial Diseases, Osaka University, Suita, Osaka, Japan; 3 Department of Food Science and Technology, College of Ocean Science and Technology, Kunsan National University, Kusan, Jeollabuk-do, Korea; 4 Centro Universitário Feevale, Novo Hamburgo, Rio Grande do Sul, Brazil; Columbia University, United States of America

## Abstract

*Vibrio parahaemolyticus* is an important pathogen causing food-borne disease worldwide. An 80-kb pathogenicity island (Vp-PAI), which contains two *tdh* (thermostable direct hemolysin) genes and a set of genes for the type III secretion system (T3SS2), is closely related to the pathogenicity of this bacterium. However, the regulatory mechanisms of Vp-PAI's gene expression are poorly understood. Here we report that two novel ToxR-like transcriptional regulatory proteins (VtrA and VtrB) regulate the expression of the genes encoded within the Vp-PAI region, including those for TDH and T3SS2-related proteins. Expression of *vtrB* was under control of the VtrA, as vector-expressed *vtrB* was able to recover a functional protein secretory capacity for T3SS2, independent of VtrA. Moreover, these regulatory proteins were essential for T3SS2-dependent biological activities, such as *in vitro* cytotoxicity and *in vivo* enterotoxicity. Enterotoxic activities of *vtrA* and/or *vtrB* deletion strains derived from the wild-type strain were almost absent, showing fluid accumulation similar to non-infected control. Whole genome transcriptional profiling of *vtrA* or *vtrB* deletion strains revealed that the expression levels of over 60 genes were downregulated significantly in these deletion mutant strains and that such genes were almost exclusively located in the Vp-PAI region. These results strongly suggest that VtrA and VtrB are master regulators for virulence gene expression in the Vp-PAI and play critical roles in the pathogenicity of this bacterium.

## Introduction

*Vibrio parahaemolyticus* is a gram-negative marine bacterium that causes acute gastroenteritis in humans associated with the consumption of raw or undercooked seafood [1], [2]. In some cases, infection by this pathogen results in primary septicemia and wound infections [3], [4]. Most of the clinical isolates of *V. parahaemolyticus* isolated from patients with diarrhea exhibit beta-hemolysis on a special blood agar plate (Wagatsuma agar), whereas environmental isolates barely do so [5]. This hemolysis is called the Kanagawa phenomenon (KP), which has been considered to be a useful marker to distinguish pathogenic from non-pathogenic strains [6], [7]. Thermostable direct hemolysin (TDH) is responsible for KP and purified TDH shows a number of biological effects, such as erythrocyte lysis, cytotoxicity and induction of fluid accumulation in an ileal loop model [6], [8], [9], [10], [11], [12], [13], [14], [15], [16], [17]. Thus, TDH has been considered a major virulence factor of *V. parahaemolyticus*.

Whole genome sequencing of a KP-positive *V. parahaemolyticus* strain RIMD2210633 revealed that this strain contains two sets of gene clusters for Type III Secretion System (T3SS), one on each of its two chromosomes (termed T3SS1 and T3SS2, respectively) [18]. Recently, comparative genomic analysis using microarray revealed that an 80-kb pathogenicity island (Vp-PAI) on chromosome II is conserved exclusively in KP-positive pathogenic strains and not in KP-negative strains [19], [20]. Vp-PAI contains not only two *tdh* genes (*tdhA* and *tdhS*) but also the T3SS2 gene cluster. This is highly associated with KP-positive strains and is also involved in the enterotoxicity of this bacterium [19], [21], [22]. Therefore, Vp-PAI has been considered to be related to the pathogenicity of *V. parahaemolyticus* in humans. Despite having an important role in pathogenicity in humans, the regulatory mechanism of genes expression from Vp-PAI is poorly understood.

In this study, we show that two putative DNA-binding proteins encoded within the Vp-PAI region, which have a winged-helix-turn-helix (WHTH) DNA-binding domain of the OmpR family, control the expression of Vp-PAI's genes in a highly specific manner. Accordingly, they must play a critical role in the pathogenicity of *V. parahaemolyticus*.

## Results

### VPA1332 (VtrA) and VPA1348 (VtrB) Have a Winged-Helix-Turn-Helix (WHTH) DNA-Binding Domain of the OmpR Family

**Figure 1:**
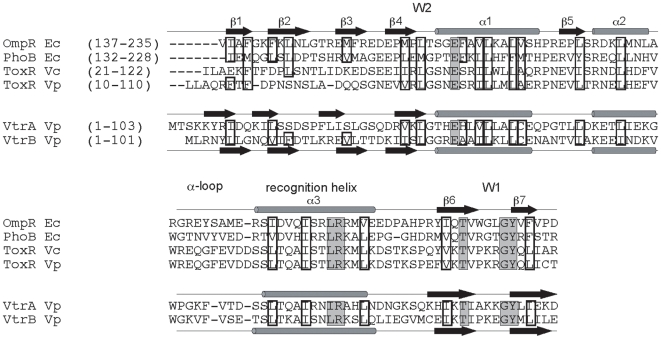
VPA1332 (VtrA) and VPA1348 (VtrB) have a winged-helix-turn-helix DNA-binding domain of OmpR family. Multiple sequence alignment and secondary structure assignments of DNA-binding and trans-activation domains of OmpR, PhoB, ToxR, VtrA, and VtrB proteins are shown. The amino acids that form the hydrophobic cores are highlighted with boxes. Highly conserved amino acids are highlighted with gray boxes.

In our functional analysis of T3SS2 in *V. parahaemolyticus*, we noted that two open reading frames (ORFs) (VPA1332 and VPA1348), which share a degree of identity with the N-terminal end of *V. cholerae* and *V. parahaemolyticus* ToxR (32% and 34% identity with *V. cholerae* ToxR, and 45% and 32% identity with *V. parahaemolyticus* ToxR, respectively), were encoded in the Vp-PAI locus. ToxR is a transcription factor found in *V. cholerae* that regulates expression of the genes encoding cholera toxin (CT) and toxin-coregulated pilus (TCP) [23]. The N-terminal domain of ToxR encodes a WHTH DNA-binding domain, which is a typical characteristic of the OmpR family of proteins and is necessary for transcriptional regulation of ToxR regulons [24]. The WHTH domain consists of an amino-terminal four-stranded beta sheet, a central three-helical bundle and a carboxy-terminal two-stranded beta sheet. The predicted secondary structures of the N-terminal portions of VPA1332 and VPA1348 were also similar to the DNA-binding domains of OmpR and PhoB of *E. coli* (Fig. 1). Multiple sequence alignments of these proteins revealed that most of the amino acids forming hydrophobic cores were conserved in VPA1332 and VPA1348 and that highly conserved amino acids were identical to that of OmpR and PhoB. Therefore, VPA1332 and VPA1348 were termed VtrA (*V. parahaemolyticus* T3SS2 regulator A) and VtrB, respectively. Their possible roles as transcriptional regulators were examined in the following experiments.

### VtrA and VtrB Regulate the Expression of the Genes for T3SS2-Related Proteins and TDH

**Figure 2:**
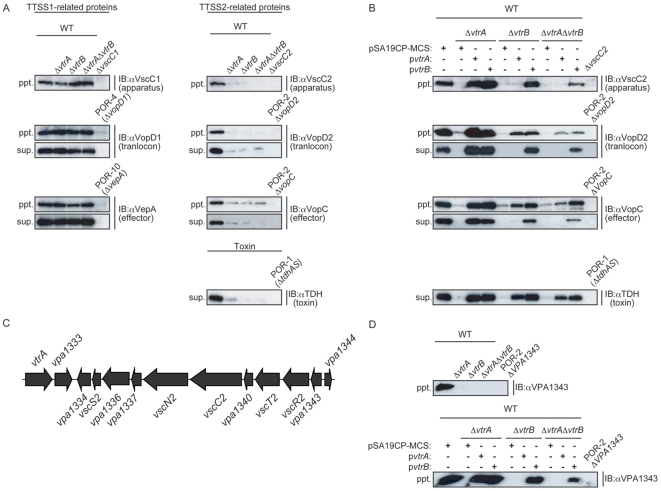
VtrA and VtrB regulate the expression levels of T3SS2-related proteins and TDH. A. Loss of *vtrA* and *vtrB* diminished the expression of T3SS2-related proteins and TDH. Western blot analysis of bacterial pellets (ppt.) and secreted proteins (sup.) from isogenic mutants of wild-type (WT) *V. parahaemolyticus*. Lane 1, wild-type *V. parahaemolyticus* (WT); lane 2, *vtrA* deletion strain (WT*ΔvtrA*); lane 3, *vtrB* deletion strain (WT*ΔvtrB*); lane 4, *vtrA* and *vtrB* double deletion strain (WT*ΔvtrAΔvtrB*). Samples from indicated strains were loaded in lane 5 to confirm the specificity of each antibody. Blots were probed with anti-VscC1, anti-VopD1, anti-VepA, anti-VscC2, anti-VopD2, anti-VopC, and anti-TDH polyclonal antibodies. B. Vector-induced *vtrB* could restore the secretory capacity of T3SS2 independent of *vtrA*. Western blot analyses of bacterial pellets (ppt.) and secreted proteins (sup.) from indicated strains are shown. Blots were probed with anti-VscC2, anti-VopD2, anti-VopC, and anti-TDH polyclonal antibodies. C. Genetic organization of the DNA region containing *vscC2* and *vpa1343* of *V. parahaemolyticus* RIMD2210633. D. VPA1343 protein expression was strictly regulated by VtrB. Western blot analysis of bacterial pellets (ppt.) from isogenic mutants of wild-type (WT) *V. parahaemolyticus* (upper panel) and their complemented strains (lower panel). Blots were probed with anti-VPA1343 polyclonal antibodies.

We first constructed *vtrA* and/or *vtrB* deletion strains from TDH-producing *V. parahaemolyticus* RIMD2210633 and then examined their effects on the production of T3SS1- and T3SS2-related proteins and TDH by immunoblotting. As shown in Fig. 2A, deletion of the *vtrA* or *vtrB* genes did not affect on the production of T3SS1-related proteins (VscC1, T3SS1 apparatus protein; VopD1, T3SS1 translocon protein and VepA, T3SS1 effector protein), whereas either deletion mutant produced a marked decrease in T3SS2-related proteins (VscC2, T3SS2 apparatus protein; VopD2, T3SS2 translocon protein VopC, T3SS2 effector protein) and in TDH both in bacterial pellets and supernatants. The amounts of T3SS2-related proteins and TDH were recovered fully in both the bacterial pellets and supernatants by complementation of each gene (Fig. 2B). Interestingly, vector-expressed *vtrB* (p*vtrB*) could also completely restore the production of T3SS2-related proteins and TDH for the WT*ΔvtrA* strain in both the bacterial pellets and supernatants. Although vector-expressed *vtrA* (p*vtrA*) could recover the production of TDH in the supernatant and VopD2 and VopC proteins of the bacterial pellet of the WT*ΔvtrB* strain, no VscC2 protein was found in the bacterial pellet, and neither of the VopD2 and VopC proteins could be detected in the supernatant. In a similar fashion, in a double deletion mutant strain (WT*ΔvtrAΔvtrB*), complementation with *vtrB* (p*vtrB*) led to recovery of all proteins in both the bacterial pellet and the supernatant, whereas VopD2 and VopC proteins in the supernatant and VscC2 protein in the bacterial pellet were not detected by complementation with *vtrA* (p*vtrA*). Unlike VopD2, VopC, and TDH, VscC2 protein production seemed to be controlled strictly by *vtrB*. Hence, it was next determined whether a gene located on the same operon as the *vscC2* gene was also regulated by VtrB. Production of the VP1343 protein, which was expected to be co-transcribed with *vscC2* (Fig. 2C), was examined by immunoblotting. VP1343 protein was not detected in bacterial pellets from *vtrA* and/or *vtrB* deletion strains (Fig. 2D, upper panel). Similar to the VscC2 protein, vector-expressed *vtrB* could overcome a defect in VPA1343 production in *vtrA* deletion strains, such as WT*ΔvtrA* and WT*ΔvtrAΔvtrB*, whereas vector-expressed *vtrA* did not induce VPA1343 production in *vtrB* deletion strains (WT*ΔvtrB* and WT*ΔvtrAΔvtrB*) (Fig. 2D, lower panel). Together, these results suggest that *vtrA* and *vtrB* are necessary for the expression of genes encoding T3SS2-related proteins and TDH and that the operon containing *vscC2* and *vpa1343* is strictly controlled by *vtrB*. As this operon contains some genes homologous to the T3SS-apparatus (*vscS2*, *vscN2*, *vscC2*, *vscT2* and *vscR2*) (Fig. 2C) that are essential for T3SS secretion, this could explain why, in vector-expressed *vtrA*, only T3SS2 secreted proteins (VopD2 and VopC proteins) in the bacterial pellets of the WT*ΔvtrB* and WT*ΔvtrAΔvtrB* strains.

### Expression of VtrB Is Controlled Directly by VtrA

**Figure 3:**
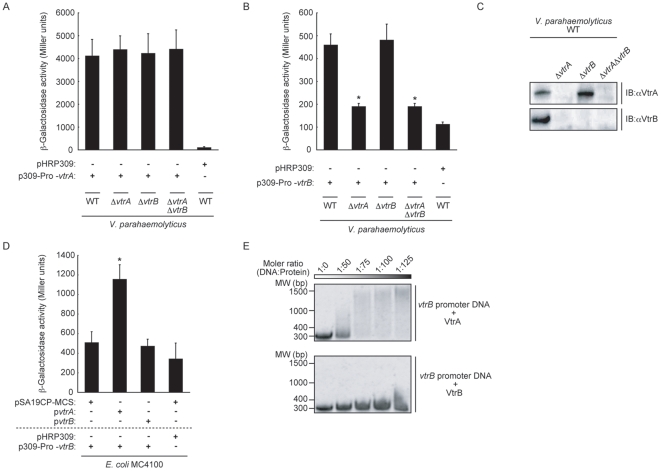
VtrB expression is under the control of VtrA. A. Neither *vtrA* nor *vtrB* was involved in the transcription of *vtrA*. *V. parahaemolyticus* strains carrying the *vtrA-lacZ* transcriptional fusion vector were assayed for β-galactosidase activity. The bars show the average of three separate experiments, and the standard deviations are indicated by error bars. B. Transcription of *vtrB* was decreased in *vtrA* deletion strains. *V. parahaemolyticus* strains carrying the *vtrB-lacZ* transcriptional fusion vector were assayed for β-galactosidase activity. The bars show the average of three separate experiments, and the standard deviations are indicated by error bars. C. Deletion of *vtrA* caused a decrease in the production of VtrB. Immunoblot analysis of VtrA and VtrB protein expression in *vtrA* and *vtrB* mutant strains are shown. Lane 1, wild-type *V. parahaemolyticus* (WT); lane 2, *vtrA* mutant strain (WT*ΔvtrA*); lane 3, *vtrB* mutant strain (WT*ΔvtrB*); lane 4, *vtrA* and *vtrB* double mutant strain (WT*ΔvtrAΔvtrB*). Blots were probed with anti-VtrA (upper panel) and anti-VtrB (lower panel) polyclonal antibodies. D. Effects of *vtrA* and *vtrB* expression on *vtrB* transcription in *E. coli*. *E. coli* MC4100 carrying *vtrB-lacZ* transcriptional fusion vector were assayed for β-galactosidase activity. The bars show the average of three separate experiments, and the standard deviations are indicated by error bars. E. Binding of purified VtrA DNA binding domain to the upstream region of *vtrB* is shown by an electrophoretic mobility shift assay. Each lane contains the same amount of upstream region of *vtrB* (30 nM) and various concentrations (0, 1.5, 2.25, 3.0, 4 µM) of VtrA DNA binding domain (upper panel) or VtrB DNA binding domain (lower panel). The molecular ratios are indicated in the top line.

Based on the above observations that vector-induced *vtrB* could restore the production of TDH and T3SS2-related protein even though both *vtrB* and *vtrA* are essential for the production of these proteins (Fig. 2), we next examined the possibility that *vtrA* might regulate *vtrB* expression. For this, we used *vtrA*-*lacZ* or *vtrB-lacZ* transcriptional fusion reporters. Neither *vtrA* nor *vtrB* gene deletion had any influence on *vtrA-lacZ* transcription in *V. parahaemolyticus* (Fig. 3A). In contrast, *vtrB-lacZ* transcription decreased dramatically in the *vtrA* deletion strains (Fig. 3B). Immunoblotting of VtrA and VtrB proteins in the *vtrA* and/or *vtrB* deletion strains revealed that VtrA protein production occurs regardless of the expression of *vtrB*, whereas deletion of *vtrA* caused a decrease in production of the VtrB protein (Fig. 3C). This transcriptional activation of VtrA against *vtrB* gene transcription was also observed in *E. coli*, as *vtrB-lacZ* transcription was significantly induced only when VtrA was produced (Fig. 3D). Direct binding of the VtrA DNA binding domain to *vtrB* promoter DNA was then examined by a gel shift assay. A shift in electrophoretic mobility of *vtrB* promoter DNA was observed at a low concentration of the VtrA DNA binding domain (Fig. 3E, upper panel), whereas only a weak shift was seen at the highest concentration of VtrB DNA binding domain (Fig. 3E, lower panel). These results indicate that VtrA activates *vtrB* gene transcription by direct binding to its promoter.

### VtrA and VtrB Play Critical Roles in T3SS2-Dependent Cytotoxicity

**Figure 4:**
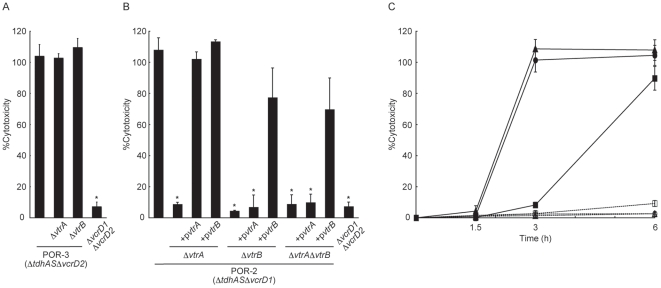
VtrA and VtrB are not necessary for T3SS1-dependent cytotoxicity but necessary for T3SS2-dependent cytotoxicity. A. *vtrA* and *vtrB* are not necessary for T3SS1-dependent cytotoxicity. Caco-2 cells were infected for 6 h with isogenic strains of POR-3 (*ΔtdhASΔvcrD2*). Bar 1: POR-3 (*ΔtdhASΔvcrD2*); bar 2: POR-3*ΔvtrA*; bar 3: POR-3*ΔvtrB*; bar 4: *ΔvcrD1ΔvcrD2* (*ΔtdhASΔvcrD1ΔvcrD2*). Cytotoxicity was evaluated by the amount of LDH released. Error bars represent standard deviations for results from triplicate experiments. B. *vtrA* and *vtrB* are essential for T3SS2-dependent cytotoxicity. Caco-2 cells were infected for 6 h with isogenic mutant strains of POR-2 (*ΔtdhASΔvcrD1*). Bar 1: POR-2 (*ΔtdhASΔvcrD1*); bar 2: POR-2*ΔvtrA* (*ΔtdhASΔvcrD1ΔvtrA*); bar 3: POR-2*ΔvtrA* expressing *vtrA* (POR-2*ΔvtrA*+p*vtrA*); bar 4: POR-2*ΔvtrA* expressing *vtrB* (POR-2*ΔvtrA*+p*vtrB*); bar 5: POR-2*ΔvtrB* (*ΔtdhASΔvcrD1ΔvtrB*); bar 6; POR-2*ΔvtrB* expressing *vtrA* (POR-2*ΔvtrB*+p*vtrA*); bar 7: POR-2*ΔvtrB* expressing *vtrB* (POR-2*ΔvtrB*+p*vtrB*); bar 8: POR-2*ΔvtrAΔvtrB* (*ΔtdhASΔvcrD1ΔvtrAΔvtrB*); bar 9: POR-2*ΔvtrAΔvtrB* expressing *vtrA* (POR-2*ΔvtrAΔvtrB*+p*vtrA*); bar 10: POR-2*ΔvtrAΔvtrB* expressing *vtrB* (POR-2*ΔvtrAΔvtrB*+p*vtrB*); bar 11: *ΔvcrD1ΔvcrD2* (*ΔtdhASΔvcrD1ΔvcrD2*). Cytotoxicity was evaluated by the amount of LDH released. Error bars represent standard deviations for results from triplicate experiments. Asterisks indicate significant differences from the results obtained with the parent strain (*P<0.05). C. Overexpressing of *vtrA* and *vtrB* promoted T3SS2-dependent cytotoxicity. Caco-2 cells were infected for 1.5–6 h with *V. parahaemolyticus*. Cytotoxicity was evaluated by the amount of LDH released. POR-2 (*ΔtdhASΔvcrD1*) with control vector (pSA19CP-MCS) (filled squares, solid line), POR-2 expressing *vtrA* (filled circles, solid line), POR-2 expressing *vtrB* (filled triangles, solid line), *ΔvcrD1ΔvcrD2* (*ΔtdhASΔvcrD1ΔvcrD2*) with control vector (pSA19CP-MCS) (open squares, dashed line), *ΔvcrD1ΔvcrD2* expressing *vtrA* (open circles, dashed line), and *ΔvcrD1ΔvcrD2* expressing *vtrB* (open triangles, dashed line). Error bars represent standard deviations for results from triplicate experiments. Asterisks indicate significant differences from the results obtained with the parent strain (*P<0.05).

One characteristic of T3SSs in *V. parahaemolyticus* is their ability to cause cytotoxic effects on Caco-2 cells *in vitro* [25], [26]. Therefore, we next examined the role of *vtrA* and *vtrB* in T3SS1- and T3SS2-mediated cytotoxicity. Deletion of the *vtrA* or the *vtrB* gene in the *tdhAS-* and T3SS2-deficient strain POR-3 (POR-3*ΔvtrA* and POR-3*ΔvtrB*, respectively) had no effect on T3SS1-dependent cytotoxicity (Fig. 4A). By contrast, deletion of the *vtrA* and/or the *vtrB* from the *tdhAS*- and T3SS1-deficient strain POR-2 (POR-2*ΔvtrA*, POR-2*ΔvtrB*, and POR-2*ΔvtrAΔvtrB*, respectively) caused a decrease in cytotoxicity similar to that of the *ΔvcrD1ΔvcrD2* strain, which is deficient in both T3SS1 and T3SS2 (Fig. 4B). A vector-expressed *vtrB* was able to overcome the defect in cytotoxicity of *vtrA* deletion strains (POR-2*ΔvtrA* and POR-2*ΔvtrAΔvtrB*). This result is in accordance with the previous results, showing that vector-expressed *vtrB* could recover the diminished secretory capacity of T3SS2 in *vtrA* deletion strains as shown in Fig. 2B and C. In contrast, complementation with *vtrA* restored cytotoxic capacity in the POR-2*ΔvtrA* strain but not in any of the POR-2*ΔvtrB* or POR-2*ΔvtrAΔvtrB* strains, which is also in agreement with the results shown in Fig. 2. Finally, the effect of *vtrA* and *vtrB* overexpression on T3SS2-dependent cytotoxicity was determined (Fig. 4C). Overexpression either *vtrA* or *vtrB* resulted in dramatic accelerations in cytotoxic activity from a *tdhAS*- and T3SS1-deficient strain (POR-2), whereas no effect was observed from overexpression in a *tdhAS*- and T3SS1/T3SS2-deficient strain (*ΔvcrD1ΔvcrD2*). These results indicate that both *vtrA* and *vtrB* are essential for T3SS2-dependent cytotoxicity.

### VtrA and VtrB Play Critical Roles in *V. parahaemolyticus*-Induced Enterotoxicity

**Figure 5:**
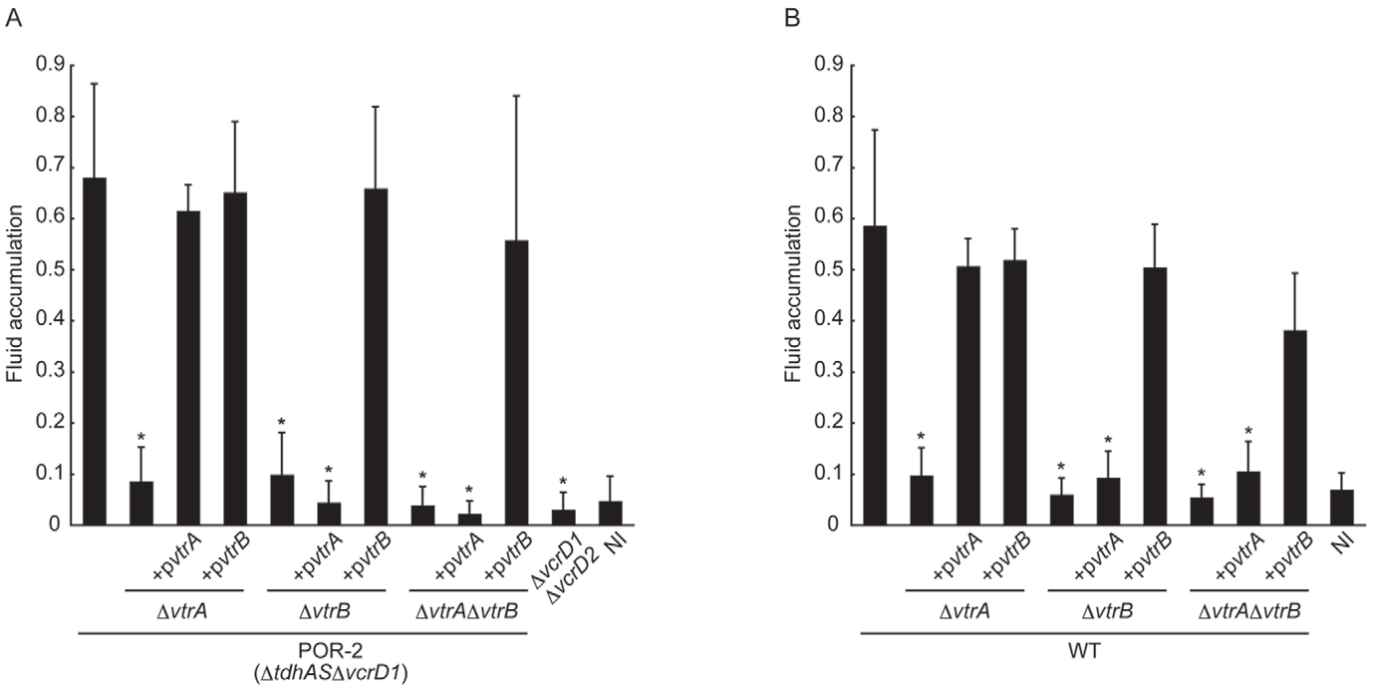
VtrA and VtrB have a critical role in *V. parahaemolyticus*-induced enterotoxicity. A. VtrA and VtrB are essential for T3SS2-dependent enterotoxicity. The enterotoxic activity levels of isogenic mutants of POR-2 (*ΔtdhASΔvcrD1*) and complemented strains in rabbit ileal loops were examined. Bar 1, POR-2 (*ΔtdhASΔvcrD1*); bar 2, POR-2*ΔvtrA* (*ΔtdhASΔvcrD1ΔvtrA*); bar 3, POR-2*ΔvtrA* expressing *vtrA* (POR-2*ΔvtrA*+p*vtrA*); bar 4, POR-2*ΔvtrA* expressing *vtrB* (POR-2*ΔvtrA*+p*vtrB*); bar 5, POR-2*ΔvtrB* (*ΔtdhASΔvcrD1ΔvtrB*); bar 6, POR-2*ΔvtrB* expressing *vtrA* (POR-2*ΔvtrB*+p*vtrA*); bar 7, POR-2*ΔvtrB* expressing *vtrB* (POR-2*ΔvtrB*+p*vtrB*); bar 8, POR-2*ΔvtrAΔvtrB* (*ΔtdhASΔvcrD1ΔvtrAΔvtrB*); bar 9, POR-2*ΔvtrAΔvtrB* expressing *vtrA* (POR-2*ΔvtrAΔvtrB*+p*vtrA*); bar 10, POR-2*ΔvtrAΔvtrB* expressing *vtrB* (POR-2*ΔvtrAΔvtrB*+p*vtrB*); bar 11, *ΔvcrD1ΔvcrD2* (*ΔtdhASΔvcrD1ΔvcrD2*); bar 12, non-infected (NI) control. Results were measured as the amount of accumulated fluid (in milliliters) per length (in centimeters) of ligated rabbit small intestine. Error bars represent standard deviations for results from triplicate experiments. Asterisks indicate significant differences from the results obtained with the parental strain (*P*<0.05). B. VtrA and VtrB are essential for *V. parahaemolyticus*-induced enterotoxicity. The enterotoxic activity of isogenic mutants of wild-type *V. parahaemolyticus* (WT) and complemented strains in rabbit ileal loops were examined. Bar 1, wild-type (WT); bar 2, WT*ΔvtrA*; bar 3, WT*ΔvtrA* expressing *vtrA* (WT*ΔvtrA*+p*vtrA*); bar 4, WT*ΔvtrA* expressing *vtrB* (WT*ΔvtrA*+p*vtrB*); bar 5, WT*ΔvtrB*; bar 6, WT*ΔvtrB* expressing *vtrA* (WT*ΔvtrB*+p*vtrA*); bar 7, WT*ΔvtrB* expressing *vtrB* (WT*ΔvtrB*+p*vtrB*); bar 8, WT*ΔvtrAΔvtrB*; bar 9, WT*ΔvtrAΔvtrB* expressing *vtrA* (WT*ΔvtrAΔvtrB*+p*vtrA*); bar 10, WT*ΔvtrAΔvtrB* expressing *vtrB* (WT*ΔvtrAΔvtrB*+p*vtrB*); bar 11, NI control. Error bars represent standard deviations for results from triplicate experiments. Asterisks indicate significant differences from the results obtained with the parental strain (*P*<0.05).

To investigate the contribution of *vtrA* and *vtrB* to the enterotoxicity of *V. parahaemolyticus*, we examined the T3SS2-dependent enterotoxic activity of the *vtrA* and *vtrB* deletion strains using the rabbit ileal loop model. As reported previously, POR-2, which is a *tdhAS*- and T3SS1-deficient strain, caused a high level of fluid accumulation [21], [26]. This was dramatically decreased in *vtrA* and/or *vtrB* deletion strains (POR-2*ΔvtrA*, POR-2*ΔvtrB*, and POR-2 *ΔvtrA ΔvtrB*) and was similar to that of the *ΔvcrD1ΔvcrD2* strain and non-infected (NI) control (Fig. 5A). The decrease in enterotoxicity was restored by trans-complementation of each gene. As with T3SS2-dependent cytotoxicity shown in Fig. 4B, a defect in enterotoxicity of *vtrA* deletion strains (POR-2*ΔvtrA* and POR-2*ΔvtrAΔvtrB*) was restored by vector-expressed *vtrB* (Fig. 5A). Similar to T3SS2-dependent enterotoxicity, both *vtrA* and *vtrB* greatly contributed to the wild-type *V. parahaemolyticus*-induced enterotoxicity, which has functional TDH and both T3SS1 and T3SS2 (Fig. 5B). The fluid accumulation that resulted after challenge with *vtrA* and/or *vtrB* deletion strains (WT*ΔvtrA*, WT*ΔvtrB*, and WT*ΔvtrAΔvtrB*) was almost none, very similar to the NI control. Vector-expressed *vtrB* was able to restore the enterotoxicity to its full potential not only in the *vtrB* deletion strain, but also in *vtrA* deletion strains. These results indicate strongly that both *vtrA* and *vtrB* play critical roles in *V. parahaemolyticus*-induced enterotoxicity.

### VtrA and VtrB Specifically Regulate Genes Encoded in the Vp-PAI Region

**Figure 6:**
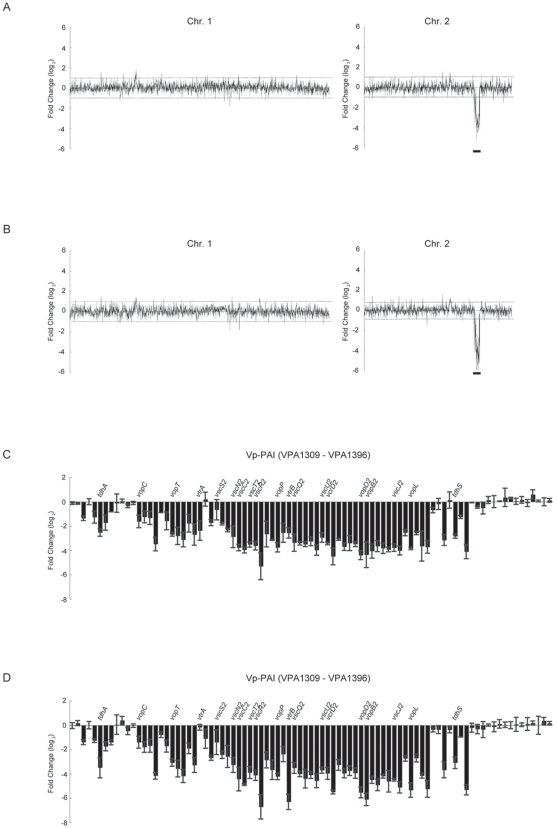
Whole-genome transcriptional profiling of *vtrA* and *vtrB* deletion strain. Genome-wide transcript analysis of the VtrA and VtrB regulons is shown. Gene expression was determined by comparing cDNA generated from WT*ΔvtrA* (A) or WT*ΔvtrB* (B) in exponential phase grown in LB medium with 0.5% NaCl with that from the WT strain. The Vp-PAI region is indicated by a bold line. Effect of the *vtrA* (C) or *vtrB* (D) deletion on expression of genes located within Vp-PAI (*vpa1309*-*vpa1396*). Representative gene functions are indicated at the top.

To identify transcriptional targets of VtrA and VtrB on the complete chromosomes of *V. parahaemolyticus*, genome-wide transcriptional profiles of *vtrA* or *vtrB* deletion strains were compared with that of the wild-type strain (Fig. 6 and Table 1). Overview of the transcriptome microarray analysis revealed that most gene expressions were unaffected by deletion of the *vtrA* or the *vtrB* genes respectively (Fig. 6A, B, respectively). However, it was also obvious that genes located on a particular region of chromosome 2 were remarkably down-regulated in both *vtrA* and *vtrB* deletion strains (Fig. 6A, B; lined). Interestingly, this region is included in the Vp-PAI region (*vpa1309*-*vpa1396*) [19], [20] and the transcription of both *tdhAS* genes and T3SS2-related genes were also decreased significantly in each of the mutant strains (Fig. 6C, D). The pattern of gene expression profile was almost similar between these two mutant strains. Expression of *vtrA* was not affected by *vtrB* gene deletion (–1.1 -fold change), which is in accordance with our previous observations that *vtrB* gene deletion did not have any effect on *vtrA* gene expression, as assessed by the reporter gene assay of *vtrA* and immunoblotting of VtrA (Fig. 3A, C). Only a few other genes encoded outside of the Vp-PAI region were affected (Table 1). These results suggest that these two proteins regulate gene expression in the Vp-PAI region in highly specific manners.

**Table 1:** Microarray analysis of VtrA and VtrB regulon in *V. parahaemolyticus*.

		Fold change^a^	
Identification	ORF Description	*vtrA*	*vtrB*
Downregulated genes (VtrA or VtrB-activated)			
VP0047	peptide ABC transporter, ATP-binding protein	−1.6	**−2.0**
VP1880	L-serine dehydratase 1	−1.4	**−2.2**
VP1904	methyl-accepting chemotaxis protein	**−3.6**	−3.6
VP2015	putative cytochrome c	−2.1	**−3.4**
VP2016	hypothetical protein	**−2.4**	−2.4
VP2362	outer membrane protein OmpK precursor	−2.0	**−2.0**
VP2679	ribosomal large subunit pseudouridine synthase A	−1.5	**−2.0**
VPA1311	hypothetical protein	−2.6	**−2.6**
VPA1313	hypothetical protein	−2.4	**−2.4**
VPA1314	thermostable direct hemolysin A	**−5.8**	**−11.3**
VPA1315	hypothetical protein	−3.3	**−3.3**
VPA1321	cytotoxic necrotizing factor	**−3.1**	−3.1
VPA1322	putative zinc finger protein	−2.4	**−3.5**
VPA1323	hypothetical protein	−2.5	**−3.2**
VPA1324	hypothetical protein	**−11.2**	**−17.8**
VPA1326	hypothetical protein	−3.0	**−3.3**
VPA1327	putative exoenzyme T	**−6.5**	**−8.4**
VPA1328	hypothetical protein	**−7.0**	**−11.9**
VPA1329	putative traA protein	**−8.7**	**−18.0**
VPA1330	hypothetical protein	−3.4	**−3.7**
VPA1331	putative OspC2	**−6.5**	**−9.6**
VPA1332	VtrA protein	−5.2	−1.1
VPA1334	hypothetical protein	**−3.3**	**−6.4**
VPA1336	hypothetical protein	**−3.5**	**−5.4**
VPA1337	hypothetical protein	**−5.0**	**−5.0**
VPA1338	putative ATPase YscN	**−7.4**	**−9.6**
VPA1339	putative type III secretion system EscC protein	**−13.7**	**−21.5**
VPA1340	hypothetical protein	**−15.5**	**−29.2**
VPA1341	putative Spa29, component of the Mxi-Spa secretion machinery	**−11.5**	**−14.8**
VPA1342	putative Type III secretion protein Spa24	**−12.3**	**−17.3**
VPA1343	hypothetical protein	**−39.7**	**−104.7**
VPA1344	hypothetical protein	−6.3	**−7.3**
VPA1345	hypothetical protein	**−8.9**	**−12.7**
VPA1346	putative targeted effector protein YopP	**−13.5**	**−18.5**
VPA1347	hypothetical protein	**−5.9**	**−5.2**
VPA1348	VtrB protein	**−5.9**	**−78.8**
VPA1349	putative Type III secretion protein Spa33	**−10.4**	**−11.4**
VPA1350	hypothetical protein	**−10.7**	**−16.0**
VPA1351	hypothetical protein	**−11.3**	**−21.0**
VPA1352	hypothetical protein	**−9.6**	**−17.0**
VPA1353	putative outer membrane protein	**−15.7**	**−23.7**
VPA1354	putative type III secretion system EscU protein	**−7.7**	**−12.9**
VPA1355	putative type III secretion system EscV protein	**−10.8**	**−15.5**
VPA1356	hypothetical protein	**−22.7**	**−44.8**
VPA1357	hypothetical protein	−8.6	**−7.3**
VPA1358	putative dimethyladenosine transferase	**−13.0**	**−15.4**
VPA1359	hypothetical protein	**−10.6**	**−13.3**
VPA1360	hypothetical protein	**−11.2**	**−15.2**
VPA1361	hypothetical protein	**−21.2**	**−46.1**
VPA1362	putative secreted protein EspD	**−20.6**	**−68.5**
VPA1363	putative chaperone	**−16.7**	**−22.5**
VPA1364	hypothetical protein	**−12.6**	**−30.0**
VPA1365	putative two-component response regulator	**−14.2**	**−18.2**
VPA1366	hypothetical protein	**−15.3**	**−24.3**
VPA1367	putative type III secretion system lipoprotein precursor EprK	**−13.8**	**−22.4**
VPA1368	hypothetical protein	**−16.2**	**−34.5**
VPA1369	hypothetical protein	**−5.8**	**−6.6**
VPA1370	hypothetical protein	**−14.4**	**−40.3**
VPA1371	hypothetical protein	**−6.0**	**−6.5**
VPA1373	hypothetical protein	**−13.2**	**−37.8**
VPA1376	conserved hypothetical protein	**−8.9**	**−12.8**
VPA1378	thermostable direct hemolysin S	**−7.2**	**−8.5**
VPA1380	putative OspB protein	**−17.3**	**−39.5**
Upregulated genes (VtrA or VtrB-repressed)			
VP0368	mannitol operon repressor	1.9	**2.1**
VP0996	putative 54 kDa polar flagellar sheath protein A	**2.0**	2.0
VPA0548	putative protein F-related protein	1.7	**2.0**

a Fold change in gene transcripts between the wild-type and *ΔvtrA* or *ΔvtrB* mutant as determined by microarray analysis. Statistically significant changes (≥2-fold difference with *P*<0.05) are highlighted in bold as described in *Materials and Methods*.

## Discussion

*Vibrio parahaemolyticus* is a gram-negative marine bacterium that causes acute gastroenteritis in humans [1], [2]. TDH has been considered a major virulence factor of gastroenteritis, because TDH is responsible for KP (a marker of pathogenic strains) and has cytotoxic and enterotoxic activities [8], [15], [27], [28], [29], [30], [31], [32]. Whole genome sequencing of this KP-positive strain revealed the presence of two sets of genes encoding for two separate type III secretion systems (T3SS1 and T3SS2)[18]. The T3SS1 gene cluster is found in both KP-negative and -positive strains, while the T3SS2 gene cluster is highly associated with KP-positive strains [21]. A functional characterization of T3SS2 has revealed that it is associated with cytotoxic activity against Caco-2 cells *in vitro* [25], [26]. Furthermore, the enterotoxicity observed for a *tdhAS* deletion mutant strain was not observed for a T3SS2-deficient mutant strain [21], [26], [33]. Therefore, T3SS2 is also thought to be related to the enterotoxicity of *V. parahaemolyticus*. Comparative genomic analysis using microarrays to analyze both pathogenic and non-pathogenic strains, revealed that only the genes in the 80-kb pathogenicity island (Vp-PAI) on chromosome II, including two *tdh* genes (*tdhAS*) and a set of type III secretion system (T3SS2), were detected only in the KP-positive pathogenic strains [19], [20]. Therefore, it has been considered that the genes encoded in the 80-kb pathogenicity island (Vp-PAI) play major roles in the pathogenicity of this bacterium. However, the regulatory mechanisms for such genes are poorly understood. In this study, we found that two novel ToxR-like transcriptional regulatory proteins (VtrA; VPA1332 and VtrB; VPA1348), which are encoded in the Vp-PAI region, played important roles in pathogenicity (enterotoxicity) of *V. parahaemolyticus*, controlling virulence genes in the Vp-PAI region (including *tdh* and T3SS2-related genes) expression. These findings strongly indicated that this pathogen equips refined virulence gene expression system to cause gastroenteritis in humans and that these regulators are key players in the virulence of this bacterium.

Recently, a T3SS2-related T3SS gene cluster was found in *trh* (TDH-related hemolysin)-positive (KP-negative) *V. parahaemolyticus* strain TH3996, which is also pathogenic to humans. These T3SS-related genes are highly associated among *trh*-positive strains [34]. The T3SS2-related T3SS gene cluster is also encoded in a flanking region of the *trh* gene on chromosome II, which is called Vp-PAI_TH3996_, and not only TRH but also the T3SS-related genes in Vp-PAI_TH3996_ are involved in the enterotoxicity of *trh*-positive strains [34]. Moreover, a T3SS2-related gene cluster was also found in non-O1, non-O139 *V. cholerae* strains, and it was required for colonization in the infant mouse model [35], [36]. Therefore, genes in the Vp-PAI region, especially those encoding for hemolysins and T3SS2, have been considered to be related to the pathogenicity of not only *V. parahaemolyticus* but also non-O1, non-O139 *V. cholerae* to humans. The T3SS gene set found in these strains contains a pair that is highly similar to the *vtrA* and *vtrB* genes, suggesting that these regulators might contribute to the virulence of these bacteria by controlling virulence gene expression levels.

The transcriptional activator ToxR controls the expression of the genes for CT, TCP, and outer membrane proteins in *V. cholerae* [23]. ToxR is an integral membrane protein and consists of three functional domains: cytoplasmic domain, transmembrane domain and periplasmic domain [37]. The N-terminal cytoplasmic domain of ToxR encodes an OmpR-like DNA-binding domain that is essential for transcriptional regulation of ToxR-regulated genes [37]. The transmembrane (TM) and periplasmic domains of ToxR are believed to act as sensors of environmental signals [38]. The N-terminal portions of VtrA and VtrB share sequence similarity with the DNA binding domain of ToxR. The TM-PRED program (http://www.ch.embnet.org/cgi-bin/TMPRED from parser; TM helix length between 17 and 33 residues; scores, >1,000) predicted that VtrA and VtrB would contain one TM region (VtrA, amino acids 134–153 and VtrB, amino acids 157–182), indicating that VtrA and VtrB are transmembrane transcriptional activators. The TM region of VtrB is located on its C-terminal end, whereas that of VtrA is located on the middle region as ToxR. Although the C-terminal region of VtrA did not show significant sequence homology with ToxR, it is possible that it might be involved in receiving and transmitting environmental signals to elicit VtrA-mediated regulation. This signal transduction could exert virulence in *V. parahaemolyticus*.

*V. parahaemolyticus* has a homolog of *V. cholerae toxRS* operon (Vp-ToxRS), and Vp-ToxR is involved in the production of TDH [39]. Recently, Nakano *et al*. reported that Hfq, which is conserved in a wide range of bacteria and modulates the stability and transcription of mRNAs, also regulates TDH expression in *V. parahaemolyticus* [40]. In our investigation, expression levels of *vp-toxR* and *hfq* were not significantly affected by deletion of *vtrA* and *vtrB* genes under our experimental conditions (GSE17242), suggesting that neither *vp-toxR* nor *hfq* is involved in TDH expression mediated by VtrA and VtrB. It is not surprising that VtrA and VtrB are regulons, because both Vp-ToxR and Hfq are global regulators and their regulons comprise many genes, including virulence-associated genes [23], [41]. Because VtrA and VtrB regulons were specifically clustered in the Vp-PAI region (Fig. 6 and Table 1), it is possible that they are more directly related to the control of pathogenicity than Vp-ToxR and Hfq.

Genome-wide transcriptional profiling of *vtrA* or *vtrB* deletion strains revealed that VtrA and VtrB regulons were specifically encoded in the Vp-PAI region (Fig. 6 and Table 1). Given our findings that the expression of *vtrB* is under control of VtrA (Fig. 3) and that vector-expressed *vtrB* could restore the defect in enterotoxicity of the WT*ΔvtrAΔvtrB* strain (Fig. 5B), VtrB might determine this specific gene expression. The G+C content of the Vp-PAI region of *V. parahaemolyticus* is lower than the average G+C content of the small chromosome (ChrII) [18], [19]. Although a consensus sequence recognized by VtrB is unknown, it is possible that this characteristic of low G+C content in Vp-PAI might be one of the factors deciding the specificity of VtrB regulons. Given that the Vp-PAI sequence is unique to KP-positive pathogenic strains, plays an important role in the pathogenicity of *V. parahaemolyticus* and that VtrB has a critical role in the expression of genes from this region, VtrB may be considered a key player in the virulence of this bacterium. Therefore, it could be an “Achilles' heel” of this pathogen. It is possible that VtrB-specific drugs would perform well in the prevention and treatment of *V. parahaemolyticus*-induced illness.

## Materials and Methods

### Bacterial Strains and Plasmids

*V. parahaemolyticus* strain RIMD2210633 (KP positive, serotype O3:K6) [18] was used for constructing deletion mutants and in functional analysis. *E. coli* DH5α and SM10*λpir* were used for general manipulation of plasmids and mobilization of plasmids into *V. parahaemolyticus*. *E. coli* MC4100 was used for reporter gene assay. The strains and plasmids used in this study are listed in Table 2.

**Table 2:** Strains and plasmids used in this study.

Strain or plasmid	Description	Source or reference
***Vibrio parahaemolyticus***		
WT	RIMD2210633 (KP positive, serotype O3:K6)	[18]
POR-1	*ΔtdhAS* derivative of WT	[21]
POR-2	POR-1 knockout of *vcrD1* (*vp1696*) gene	[21]
POR-3	POR-1 knockout of *vcrD2* (*vpa1355*) gene	[21]
*ΔvcrD1ΔvcrD2*	POR-1 knockout of *vcrD1* and *vcrD2* gene	[25]
WT*ΔvtrA*	KXV237 knockout of *vtrA* (*vp1332*) gene	This study
WT*ΔvtrB*	KXV237 knockout of *vtrB* (*vp1348*) gene	This study
WT*ΔvtrA ΔvtrB*	KXV237 knockout of *vtrA* and *vtrB* gene	This study
POR-2*ΔvtrA*	POR-2 knockout of *vtrA* (*vp1332*) gene	This study
POR-2*ΔvtrB*	POR-2 knockout of *vtrB* (*vp1348*) gene	This study
POR-2*ΔvtrA ΔvtrB*	POR-2 knockout of *vtrA* and *vtrB* gene	This study
POR-3*ΔvtrA*	POR-3 knockout of *vtrA* (*vp1332*) gene	This study
POR-3*ΔvtrB*	POR-3 knockout of *vtrB* (*vp1348*) gene	This study
*ΔvscC1*	POR-1 knockout of *vscC1* (*vp1696*) gene	[21]
POR-4	POR-1 knockout of *vopD1* (*vp1656*) gene	[43]
POR-10	POR-1 knockout of *vepA* (*vp1680*) gene	[43]
*ΔvscC2*	POR-1 knockout of *vscC2* (*vpa1339*) gene	[21]
POR-2*ΔvopD2*	POR-2 knockout of *vopD2* (*vp1361*) gene	[26]
POR-2*ΔvopC*	POR-2 knockout of *vopC* (*vpa1321*) gene	[25]
POR-2*Δvpa1343*	POR-2 knockout of *vpa1343* gene	This study
***Escherichia coli***		
DH5α	F^−^ Φ80Δ*lac*ZM15 *Δ* (*lacZYA argF*)*U169 deoP recA1 endA1 hsdR17* (r_K_^−^ m_K_^−^)	Laboratory collection
SM10λ*pir*	*thi thr leu tonA lacY supE recA*::RP4-2-Tc::Mu λ*pir* R6K	[24]
MC4100	F^–^ *araD*139 *Δ* (*argF*-*lac*) U169 *rps*L150 (Str^r^) *relA1 flbB5301 deoC1 ptsF25 rbsR*	[44]
**Plasmid**		
pHRP309	*lacZ* transcriptional fusion vector, Gm^r^	[45]
p309-Pro-*vtrA*	Derivative of pHRP309, containing *vtrA* promoter	This study
p309-Pro-*vtrB*	Derivative of pHRP309, containing *vtrB* promoter	This study
pYAK1	R6K-*ori* suicide vector containing *sacB* gene	[46]
pYAK1-*ΔvtrA*	Derivative of suicide vector pYAK1 for generating the *vtrA* deletion mutants	This study
pYAK1-*ΔvtrB*	Derivative of suicide vector pYAK1 for generating the *vtrB* deletion mutants	This study
pYAK1-*Δvpa1343*	Derivative of suicide vector pYAK1 for generating the *vpa1343* deletion mutants	This study
pSA19CP-MCS	Complement vector for *V. parahaemolyticus*, Cm^r^	[47]
p*vtrA*	Derivative of pSA19CP-MCS, containing *vtrA* gene	This study
p*vtrB*	Derivative of pSA19CP-MCS, containing *vtrB* gene	This study

### RNA Isolation

Bacterial strains were grown at 37°C in LB broth containing 0.5% NaCl to an OD_600_ of 1.0. Bacteria were harvested by centrifugation and the bacterial pellet was suspended with TRIzol Reagent (Invitrogen). After 1 h incubation at 4°C, one-fifth volume of chloroform was added to the suspension followed by recentrifugation. The aqueous layer was removed and a one-tenth volume of 3 M sodium acetate (pH 5.9) was added. Nucleic acids were precipitated with isopropanol and pelleted by centrifugation. The pellet was washed with 80% ethanol. Contaminating genomic DNA was removed from the RNA samples using Turbo DNA-free kits (Ambion). RNA was purified by acid phenol-chloroform extraction and ethanol precipitation. Finally, highly pure total RNA was further isolated using QIAGEN RNeasy Mini kits, according to the manufacturer's protocol.

### DNA Microarray

A total of 20 µg of RNA was transcribed to DNA and labeled with aminoallyl dUTP using reverse transcriptase (Superscript III; Invitrogen) and random hexamers (TAKARA Bio) as primers. The aminoallyl-labeled DNA was purified by phenol chloroform extraction and ethanol precipitation. Precipitated DNA was resolved in 50 mM NaHCO_3_ (pH 9.0) and Cy3 or Cy5 monofunctional dye (GE Healthcare) was added to the solution. After 1 h incubation, unincorporated dye was removed using CentriSep spin columns (Princeton Separations, Inc.). Hybridization and detection of microarray signals was performed as described [19]. Equal volumes of Cy3- or Cy5-labeled probes from wild type and WT *ΔvtrA* or WT *ΔvtrB V. parahaemolyticus* strain were mixed with in hybridization solution (5×SSC buffer, 0.5% SDS, 0.1 mg/ml human Cot-1 DNA). Mixtures were heated for 5 min at 95°C, followed by ice incubation. The probe mixtures were applied to a microarray slides and covered with MAUI AO lids (BioMicro Systems). Microarray slides were incubated for 16 h at 55°C in a MAUI hybridization chamber. After hybridization, the microarray slides were washed and scanned using a Scan Array Express Lite (Perkin Elmer Life and Analytical Sciences). Each experiment was repeated in triplicates. Microarray data were analyzed using ScanArray Express software (Perkin Elmer Life and Analytical Sciences). The genes regulated by *vtrA* or *vtrB* were defined as genes that exhibited at least 2-fold difference on WT*ΔvtrA* or WT*ΔvtrB* in three experiments. All data were filtered for statistical significance (*P*<0.05) using *t*-tests in MultiExperiment Viewer (http://www.tm4.org/mev.html). Array results are available at the NCBI Gene Expression Omnibus database (GEO; http://www.ncbi.nlm.nih.gov/geo/) under the accession number GSE17242.

### Immunoblot Analysis

*V. parahaemolyticus* strains were grown overnight in LB broth with 0.5% NaCl. Cultures were then diluted 1∶100 into LB broth with 0.5% NaCl and grown with shaking at 37°C for 5 h. After incubation, bacterial cultures were centrifuged and bacterial pellets solubilized with Laemmli buffer. Secreted proteins were harvested by precipitation with cold trichloroacetic acid to a final concentration of 10% (v/v) on ice for 60 min, followed by centrifugation at 48,000 *g* for 60 min. The pellets were rinsed in cold acetone and then solubilized in Laemmli buffer.

Samples for western blot analysis were separated by SDS–PAGE (10%, 10–20%, or 15–25% gradients of polyacrylamide; COSMO BIO). The transferred membrane was probed with anti-VscC1, anti-VopD1, anti-VepA, anti-VscC2, anti-VopD2, anti-VopC, anti-TDH, anti-VPA1342, anti-VtrA, or anti-VtrB rabbit polyclonal antibodies and then probed with horseradish peroxidase-conjugated goat anti-rabbit antibody (ZYMED). The blots were developed using enhanced chemiluminescence (ECL) western blotting kits (GE healthcare).

### Reporter Gene Assays

*E. coli* MC4100 or *V. parahaemolyticus* strains, each harboring a reporter plasmid were grown for 1 h at 37°C in LB broth containing 1.0 or 0.5% NaCl. β-galactosidase activity was assayed in cell lysates by Miller's method using *o*-nitrophenyl-β-D-galactopyranoside (ONPG) as a substrate [42].

### Electrophoretic Mobility Shift Assay (EMSA)

The promoter region of *vtrB*, containing a 284 bp upstream sequence of the start codon, was amplified by polymerase chain reaction (PCR). PCR products purified from agarose gels were then mixed with increasing concentrations of the purified DNA binding domains of VtrA (amino acids 1–133) and VtrB (amino acids 1–158) in a reaction buffer containing 10 µg/ml of bovine serum albumin (BSA). After 30 min incubation at room temperature, samples were separated by 5% polyacrylamide nondenatureing gels in TAE buffer at room temperature. DNA was stained with SYBR Green I Nucleic Acid Gel Stain (Lonza) and visualized with a LAS-4000 mini EPUV (Fujifilm) at 460 nm emission wevelength.

### Cytotoxicity Assays

T3SS1 and T3SS2-dependent cytotoxicity assays were performed as described [25]. Briefly, Caco-2 cells were seeded at 3×10^4^ cells per well in 96-well plates and cultured for 48 h to confluency. The cells were co-cultured for 1.5–6 h with phosphate buffered saline (PBS)-washed bacteria at a multiplicity of infection (MOI) of 10. The release of lactate dehydrogenase (LDH) into the medium was quantified using CytoTox96 (Promega). The LDH release (percent cytotoxicity) was calculated using the following equation: (optical density at 490 nm [OD_490_] of experimental release – OD_490_ of spontaneous release)/(OD_490_ of maximum release – OD_490_ of spontaneous release)×100. Spontaneous release was taken to be the amount of LDH released from the cytoplasm of uninfected cells, whereas the maximum release was the amount released by total lysis of uninfected cells.

### Rabbit Ileal Loop Test

*V. parahaemolyticus* strains were grown overnight in LB broth with 3% NaCl. Cultures were then diluted 1∶100 into LB broth with 3% NaCl and grown with shaking for 5.5 h. After incubation, bacteria were harvested by centrifugation and suspended in LB broth with 0.5% NaCl. The bacterial suspensions (10^9^ CFU) were injected into the ligated ileal loops of rabbits, and fluid accumulation in each loop was measured at 16 h after challenge. The result was expressed as the amount of accumulated fluid (in milliliters) per length (in centimeters) of ligated rabbit small intestine. All animal experiments were performed according to an experimental protocol approved by the Ethics Review Committee for Animal Experimentation of Research Institute for Microbial Diseases (Osaka University, Osaka, Japan).

### Statistical Analysis

All data are presented as the mean and standard deviation of three determinations per experimental condition. The statistical significance was determined by one-way ANOVA followed by Dunnett's multiple comparison test, and *P*<0.05 was considered statistically significant.
